# Typhoid Fever and Epidemic Dysentery

**Published:** 1861-07

**Authors:** 


### EDITORIAL AND MISCELLANEOUS.

### Typhoid Fever and Epidemic Dysentery.

It is a fact, which has perhaps been observed by most practitioners, that Dysentery, occurring while an epidemic of this disease prevails, though with symptoms no more violent at first than is met with in the most ephemeral form, often resists the ordinary treatment, and, assuming malignant symptoms, continues for weeks, if the system of the patient is sufficiently strong to bear up under its violence. In such cases it has been observed that supporting and invigorating treatment is that which gives the only hope of preserving life in many cases. It is, under such circumstances, a disease adinamic in its character and tendencies, and does not admit of antiflogistic or defibrinating treatment. On the contrary, that which will enrich the blood and give the greatest amount of energy to the nervous system, is most useful; such as nourishing, digestible, and fluid diet, and opiate and alcoholic cerebral stimulants.

In these particulars, there is a striking similarity in the two diseases which head this article; though pathologically considered, they may differ widely from each other, yet in their practical bearing is found a striking resemblance. Indeed, in the use of astringents, which are indispensible in many cases of both diseases, an identity of treatment is observed. From these considerations the term "typhoid dysentery" would seem not inappropriately applied to epidemic dysentery. And is it unreasonable to suppose that the union of the causes of the two diseases exist in the production of this form of dysentery? While the causes which lead to the production of inflammation in the colon and rectum are in operation, the influences brought to bear in the production. typhoid fever may exist. It is not unreasonable to suppose that other diseases beside dysentery are often impressed with the adinamic character of this fever, from the existence and operation of their causes at the same time. When such depressing or adinamic influence has been exerted upon the brain, other concomitant diseases, though usually properly treated by depressing agents, require supporting remedies. The impress thus made upon ordinary inflammatory disease by atmospheric contamination, is strikingly exhibited in all diseases occurring in malarial districts. When malarial effluvia is abundantly diffused in a particular locality, the treatment of all diseases must, to some extent, be modified to meet the malarial impression made upon such cases.

